# Engaging rural preceptors in new longitudinal community clerkships during workforce shortage: a qualitative study

**DOI:** 10.1186/1471-2296-12-103

**Published:** 2011-09-27

**Authors:** Judith N Hudson, Kathryn M Weston, Elizabeth A Farmer

**Affiliations:** 1 Graduate School of Medicine, University of Wollongong, Wollongong, NSW, Australia

**Keywords:** community-based education, longitudinal clinical clerkships, medical education, rural health, preceptor recruitment, workforce maldistribution, qualitative research

## Abstract

**Background:**

In keeping with its mission to produce doctors for rural and regional Australia, the University of Wollongong, Graduate School of Medicine has established an innovative model of clinical education. This includes a 12-month integrated community-based clerkship in a regional or rural setting, offering senior students longitudinal participation in a 'community of practice' with access to continuity of patient care experiences, continuity of supervision and curriculum, and individualised personal and professional development. This required developing new teaching sites, based on attracting preceptors and providing them with educational and physical infrastructure. A major challenge was severe health workforce shortages.

**Methods:**

Before the new clerkship started, we interviewed 28 general practitioners to determine why they engaged as clerkship preceptors. Independent researchers conducted semi-structured interviews. Responses were transcribed for inductive qualitative content analysis.

**Results:**

The new model motivated preceptors to engage because it enhanced their opportunities to contribute to authentic learning when compared with the perceived limitations of short-term attachments. Preceptors appreciated the significant recognition of the value of general practice teaching and the honour of major involvement in the university. They predicted that the initiative would have positive effects on general practitioner morale and improve the quality of their practice. Other themes included the doctors' commitment to their profession, 'handing on' to the next generation and helping their community to attract doctors in the future.

**Conclusions:**

Supervisors perceive that new models of clinical education offer alternative solutions to health care education, delivery and workforce. The longitudinal relationship between preceptor, student and community was seen as offering reciprocal benefits. General practitioners are committed to refining practice and ensuring generation of new members in their profession. They are motivated to engage in novel regional and rural longitudinal clinical clerkships as they perceive that they offer students an authentic learning experience and are a potential strategy to help address workforce shortages and maldistribution.

## Background

Many countries are experiencing medical workforce shortage, particularly in rural and remote settings [1,2]. One strategy to address this, an increased number of university places for medical students, has prompted reflection on clinical education [3]. How and where best to educate these students to produce skilled graduates and address workforce maldistribution?

Clinical education, a core component of undergraduate medical education, usually comprises early introductory clinical experiences and clerkships in the senior year(s). Clerkships have typically provided a series of short-term rotations through mainly hospital-based specialities. The medical student is supervised by a range of clinical supervisors, and encounters only patients who happen to receive acute care from those services. There is a growing sense that a training model consisting of random and short-term opportunistic in-patient care experiences is poorly aligned with student learning, and current or future health care needs [4,5].

This concern has driven a series of reforms in the clinical training of medical students especially over the past decade, resulting in a trend for longitudinal rather than short-term clerkships and an increased emphasis on community-based medical education. The case for community-based education includes factors such as the change in case-mix and decrease in hospital admissions; the opportunity for health promotion and disease prevention in community settings; the value of continuity of care and chronic illness experiences for student learning; and the challenge of managing increasing student numbers [6]. As a result, some medical schools internationally are placing more focus on community-based medical education, attaching students to a community-based general/family practice [7-9].

Informed by evidence from these reforms in clinical education and in keeping with its mission to produce doctors for rural and regional Australia, the University of Wollongong, Graduate School of Medicine has recently established an innovative model of clinical clerkship. This involves a 12-month integrated community-based clerkship where senior medical students in the third year of their 4-year course live, learn and work in a regional or rural community hub. Some students with special circumstances (e.g. health or personal reasons) are placed in the regional setting (a non-capital city metropolitan area) with the majority experiencing the clerkship in small (population 5 to 8, 000) or large (population 8 to 30, 000) rural communities.

The Parallel Rural Community Curriculum Model, implemented in several small sites in rural South Australia [7,10], has been adopted as a pedagogical approach and modified as needed for each of the ten learning hubs. Each hub offers students a base in general practice and a range of clinical learning experiences including local hospital emergency and ward-based care, community health, aged-care and consultations supervised by resident or visiting specialists.

This model of education is informed by the social learning theory, 'communities of practice', which is a broad conceptual framework for thinking about learning as a process of social participation [11]. Wenger's community of practice theory provides a framework to appreciate how academic health care communities of practice can facilitate student learning. Communities of practice (CoPs) such as these have three fundamental elements: a domain of knowledge that creates a common ground and a sense of common identity; a community of people who care about the domain and create the social fabric of learning; and a shared practice that the community develops to be effective in their domain [12].

Medical students are newcomers who are included in the CoP by a process called legitimate peripheral participation [11,13]. Peripherality, an approximation of full participation, gives exposure to actual practice. Students under close supervision are part of, not outside of, practice. In the words of one clinical supervisor, the curriculum...... *just walks and wheels itself in the door*. Participation in these clinical experiences largely determines the curriculum and is key to student learning in this model. Observation can be useful but only as a prelude to actual engagement [11].

Newcomers such as senior students must be granted enough legitimacy to be treated as potential members of a health care team otherwise they are unlikely to meet the standards that the community regards as competent engagement. The supervisor's standing in the community gives the student legitimate entry to practice and being a legitimate member gives the student supervised access to CoP's learning opportunities. Peripherality and legitimacy are achievements that involve negotiation between both a CoP and its newcomer(s), and year-long placement allows time for this to be achieved. Rather than the master/student relationship of the traditional apprenticeship, this model allows a student to experience increasing participation and identity transformation over time in supportive CoPs.

Longitudinal placements in undergraduate medical education are developing and thriving internationally. A recent survey of sixteen medical schools from four countries revealed that the sub-group of 2, 700 students who had completed longitudinal integrated clerkships were very supportive of the program, and performed as well as colleagues from more traditional clerkships in national exams [14]. Other student outcomes reported from national and international long-term placements have included continuity of patient care experiences; continuity of supervision and curriculum; advocacy for the patient and health service; participatory learning; mentoring; team work; and confident and skilled students [4,15,16].

However it is a daunting challenge to expand a longitudinal curriculum to an entire rather than a sub-group of a medical school cohort, especially at a time of growing student numbers, implementation of new medical schools and the ongoing competition for teachers among medical schools [17]. Hemmer argues that moving education out of the traditional tertiary hospital would mean students wouldn't get to work with the most gifted, senior teachers [17]. However, the increase in medical student numbers has not been matched by an increase in numbers of senior hospital clinicians, and the alternative thesis is that now is the time to harness the proven skills and expertise already in the community medical arena in postgraduate education and to develop this newer group of teachers in delivering innovative undergraduate education.

Hemmer also raises the important issue of financially supporting the teachers with longitudinal programs, a difficult task for an entire cohort of students [17]. Community-based teaching would be required to extend beyond a single institution to multiple teaching sites in primary care practices, hospitals, and other health care centres. Hemmer advises that the task for clerkship directors of simultaneously monitoring an integrated, longitudinal clerkship program for an entire class across multiple sites would be momentous.

This challenge however was taken up by the University of Wollongong in July 2009. As the length of residence in a rural area is the strongest predictor of intention to take up rural medical practice [18], and the length of time students spend in a rural training environment appears to be one of the greatest influences on the number of general practitioners (GPs) working in rural areas [19], the longitudinal clerkships were implemented in ten teaching and learning hubs in regional and rural New South Wales. By integrating clinical education with health care in communities with workforce shortages we aimed to provide a quality clinical clerkship experience for all students in the first cohort (n = 68) and enhance the 'rural return' of these students following qualification as a clinician. Just prior to commencement of the first cohort, we asked a sample of our supervisors what motivated them to engage with this model of clinical clerkship.

This led to the following research question:

Why do rural and regional general practitioners (GPs) engage in new longitudinal integrated community-based clerkships during workforce shortage?

## Methods

A qualitative study using semi-structured interviews was conducted just before the first student cohort started their longitudinal clerkship. Ethics approval was obtained from the University of Wollongong Human Research Ethics Committee. Thirty-four medical practices which provide ambulatory primary care services in their community and who had already agreed to host a student for a 12-month placement were invited to contribute to the study to explore reasons for engagement in the initiative. Independent research officers conducted 28 semi-structured interviews with GP supervisors, from the 26 practices who consented to take part (response rate 76%). Of the participants, four (14%) had been in general practice less than 10 yrs, eleven (39%) for 10 to 20 years, and thirteen (47%) greater than 20 years. Interviews (approximately 45 minutes each) were recorded and transcribed. Identifying information was removed. Using inductive qualitative content analysis [20], two researchers (JNH, KMW) read and coded the transcripts independently and after discussion reached consensus on identified categories and clustering of these categories into three themes. We describe these themes and categories below, with illustrative citations from the interviews.

## Results

The thirteen categories identified from the content of the interviews were clustered into the following three themes: engaging in and contributing to learning; refining practice and ensuring generation of new members; and engaging in joint enterprise with the educational institution.

### 1. Engaging in and contributing to learning

On the eve of commencement of the new workforce and learning strategy, participants were positive about engagement and how they could contribute. They were also mindful of the responsibility of assuming a long-term supervisor role and the potential financial impact. The following categories were identified:

*• The longitudinal clerkship model*

Supervisors expressed confidence in the innovative clerkship, offering students access to a population of patients and an opportunity to participate in their care.

They've really tried to do something that's going to work... it's quite exciting

Well they get a chance to see the whole population versus a very small number of very sick patients

We give them [the students] a view of the health system as it works...They'll feel like they belong and...they'll be part of a team

The longitudinal feature was clearly a motivating factor with some reflecting on their own experience in short-term hospital placements as a medical student.

*My memories, when I was a student, is you never saw anyone for more than the week that they were in hospital, and you never knew what happened to them*.

Prior experience of hosting short-term students in their practice made the longitudinal feature of the model attractive to many respondents.

*Being longer is much better than a short-term placement...it's hard to get value out of a two-week placement and I really think that continuity which is something that is going to be very beneficial. The fact that it's a longer placement meant we will probably get better systems in place*.

*• Authentic and 'whole life cycle' learning from community-based education*

The clerkship based in a general practice setting was thought to offer a holistic education for students, to learn through practice rather than learning about practice.

*This is the coal face as it were for patients, for them to learn that it's just not the knowledge in the books but how do they put it in to practice...what you are talking biosocial, pathological, the whole thing rolled into one, as a holistic sort of a thing they should learn*.

Supervisors raised the idea that by allowing the student entry into and participation in their world of practice, practice itself would be a curriculum, offering access to whole life cycle care.

*We've got a huge range of medicine that just walks and wheels itself in the door. People talk about cradle to grave but we actually run from before you're born til after you're dead*.

*• A multi-disciplinary community of practice, including patients*

A lot of the community liked the idea of being involved in teaching and contributing to the greater good of our little town

I think there is a huge benefit for the medical student...exposure to lots of different GPs plus the allied health...and the nursing staff

*• A workforce strategy for their community*

Many doctors were committed to assisting with workforce recruitment for their community.

Our chief concern is manpower, medical manpower. Our practice is likely to close in five to ten years if we don't have new doctors coming

*• Responsibility for one academic year*

Participants acknowledged potential responsibilities and burden. Some felt responsible for the 'whole curriculum' and were concerned about the long time frame if it wasn't successful.

My biggest concern... is to make sure we are able to cover everything

If it isn't working out, it's a long time to keep struggling through it

*• Financial impact*

Supervisors had mixed feelings about the financial burden of engagement. It was perceived to be potentially cost-neutral, cost-positive or cost-negative. While some were unconcerned with the financial impact, most agreed that the practice infrastructure payment was influential in their engagement. Infrastructure funds allowed practice modifications such as providing a room for the student to consult independently. The following quotes illustrate a range of perspectives from participants:

○ Lack of concern

I don't go into it with the thought that the benefit is financial, it's more to do with the benefit of the students getting a sense of working in general practice which may in fact benefit us long term in terms of students coming back once they're graduates

○ Expectations of neutral financial impact

I'm hoping that this will come out roughly neutral...we should be doing it...it's nice to have a financial reward but it shouldn't be everything

○ Expectations of positive financial impact

As their skills increase I imagine it'll be financially of greater benefit to us

○ Expectations of negative financial impact

They'll slow me down. There will be a financial loss

○ Impact of the infrastructure payment

From a purely financial point of view, the grant for capital works has been necessary to improve our practice in order to take on the medical students

### 2. Refining practice and ensuring generation of new members

Many supervisors raised the issue of their responsibility to the profession: to train the next generation of doctors; pay-back for their own education; and raise professional standards. They believed that being a teaching practice raised the status of the practice, from their own and the patients' perspective. They predicted a positive impact on GP morale and that being a medical educator as well as a general medical practitioner could regenerate enthusiasm for their profession. In this cluster, four categories of responses were identified:

• Professional obligation to next generation

*We regard it as a professional privilege and duty to be involved in teaching. It's part of a tradition among medical practitioners that goes back to Hippocrates... the default position for most medical practitioners is - I ought to be doing this anyway*.

• Pay back for own education

It's a good thing to do in terms of giving something back to the profession and to the community

*• Raising professional standards*

It tends to raise the bar as far as the teachers themselves. You're called to question more... it's also a cause for reflection and possibly some updating

*• Effect on GP morale and status*

Think it is also lifts the profile of the practice. I feel positively about the fact that we can say we are a teaching practice and patients generally respond positively to that

It adds an element of different interest in the profession...you can actually make it exciting again

### 3. Engaging in joint enterprise with the educational institution

Participants welcomed the opportunity to be part of a larger CoP, which included the University, to be valued by this organisation and predicted a two-way flow of learning. The student would learn in the CoP but contribute learning as well. Supervisors wished to engage with the University, as well as the profession, to raise the standard of medical practice, and generate new medical workforce in areas of need.

*• Valued by the organisation*

So for the first time in our region, general practitioners have had this kind of honour of being given the opportunity to be significantly involved in the life of a medical school

*• Reciprocal learning*

I'm going to learn from the students just as much as they can learn from me

*• The joint enterprise*

Raise the level of standard of practice of medicine

A feeling of professional support to the University and to the profession itself to assist with the training of...junior colleagues coming through the ranks

## Discussion

Recruitment and retention of clinical teachers is a key issue when considering operating, sustaining and expanding a longitudinal clerkship model. Despite the pressures of workforce shortage, GPs participating in the study exhibited a strong professional disposition through an expressed commitment to the profession, professional standards and practice, being a member of a learning community that includes patients, health care colleagues and learners, and engaging with the University to meet the health care needs of their community. The central tenets of the clerkship model, namely longitudinal, community-based and participatory learning were strong motivations to engage.

Participants identified potential responsibilities and burden associated with the supervisor role. Previously studied supervisors reported that the magnitude of the reward exceeded the logistical and time challenges of long-term clerkships [21]. We found that our 'supervisors-to-be' were prepared to engage with the initiative whatever the financial or other impact on their practice. They predicted it would be cost-effective, cost neutral, or even cost-negative. This is in keeping with reported perceptions of rural supervisors surveyed in Australia in 2005 [22]. Many practitioners acknowledged that the small infrastructure grant provided by the University enabled them to make practice improvements and while this had facilitated their engagement, most were motivated by their love of teaching and commitment to educating future clinicians. As one of the significant pressures for rural practitioners is time management, they predicted that the longitudinal experience may be less of a time burden than a series of short-term clerkships. This was attributed to the increasing level of participation of a student who spent time in their CoP.

The study sample did not include all supervisors and practices who engaged with the initiative for the first cohort of senior students. Practices in regions where the medical school was in formal collaborations with other institutions for student placements were excluded from the sample for pragmatic reasons. However, the sample included most other practices and involved a range of practice models operating in rural and regional New South Wales, and data saturation was reached with the sample.

Further exploration of the impact of long-term integrated community-based placements on all stakeholders is warranted. While many of the studies completed to date have considered the impacts on supervisors and students, patients' perspectives need further exploration. Preliminary work has revealed that regional and rural patients are willing participants and that they are currently underutilised partners in community-based medical education [23]. Further study is underway to determine if, and why, patients are willing to accept long-term students as peripheral legitimate participants in their health care teams.

There are several other arms to the extensive study of how longitudinal integrated clerkships impact on all stakeholders. The financial impact on the practice is currently under investigation to test the assertion that where students have been actively involved in all aspects of the practice for at least 5 months, students appear to have positive effect on GP productivity without any loss in patient satisfaction [24]. Several of the practices involved in this qualitative study have given consent for inclusion in a concurrent financial impact study, and then post-placement analysis of what actually happened during the first academic year. Further work includes exploration of the process of developing and establishing the clerkships, with perspectives from students as well as preceptors.

A vital factor in further recruitment and sustainability of this clerkship model is the proportion of students who choose to return to work in rural and regional areas. Considerable success in terms of 'rural return' has been achieved by the longitudinal Rural Physician Associate Program (RPAP) addressing shortage of primary care physicians in rural Minnesota [25]. The Australian Medical Schools Outcomes Database will provide future data to gauge the success of rural return for our program.

It is evident that regional and rural clinical supervisors perceive that new models of clinical education offer alternative solutions to health care education, delivery and workforce. One supervisor, explaining his engagement in the initiative, stated that:

My accountant said GPs are serial altruists

However, altruism alone will not sustain the engagement of clinicians in student teaching and learning. With a new group of teachers taking on a significant responsibility in undergraduate clinical education, identifying motivating factors, strong educational support and development, and recognising contribution are crucial to enhance ongoing levels of participation.

To recruit more family medicine practitioners, especially those in the earlier years of their career, may require greater subsidies for undergraduate teaching, an issue that requires urgent review.

## Conclusions

General practitioners are committed to refining practice and ensuring generation of new members in their profession. They are motivated to engage in novel regional and rural longitudinal clinical clerkships as they perceive that they offer students an authentic learning experience and are a potential strategy to help address workforce shortages and maldistribution.

## Competing interests

The authors declare that they have no competing interests.

## Authors' contributions

JNH, KMW and EAF designed and managed all aspects of the research and supervised the collection of the qualitative data. JNH and KMW did most of the analysis of the data, assisted by EAF. JNH wrote the first draft of the paper and contributed to later drafts. KMW and EAF contributed to later drafts of the paper. All authors approved the final version of the paper.

## Pre-publication history

The pre-publication history for this paper can be accessed here:

http://www.biomedcentral.com/1471-2296/12/103/prepub
